# The Primary Suture of Wounds

**Published:** 1919-05-24

**Authors:** 


### WAR AND SURGICAL PROGRESS.

### I.—The Primary Suture of Wounds.

To those who visited any large number of Allied hospitals and casualty clearing-stations at different periods of the War, one of the most striking observations lay in the many and varied methods by which war wounds were being treated. The Carrel technique with its careful laboratory control and use of relatively strong antisepsis was found on the one hand; on the other, immediate removal of damaged tissue and primary suture. Between the two ranged a great number of methods and agents, all claiming more or less satisfactory results. Broadly speaking, in 1917-1918 opinion appeared to favour the view that mainly aseptic, and not antiseptic, surgery should be practised, the first in the great majority of cases, the second associated with it in those instances deemed by past experience to require the double method. At all times, however, every procedure has had both its ardent supporters and its bitter critics, and the more the hospitals visited, the more convincingly apparent it became that the results obtained depended far more upon the surgeon himself than upon the particular agent employed. Power of judgment in selecting the methods appropriate to any particular instance gave the true measure of his skill.

### Primary Suture.

Take, in the first place, what is now the great aim in this branch of surgery—namely, primary union by suture after complete operative removal of *débris* and thorough purification of the wound. Such procedure may be divided into "immediate primary suture," "delayed primary suture" where the wound is closed a day or two after its infliction, and "secondary suture" where a varying period of antispetic cleansing intervenes.

Sir A. Bowlby, in his address on "Primary Suture of Wounds," before the Royal Society of Medicine in 1918, gave an excellent review of the succeeding phases of wound treatment up to that time. Until the middle of 1915 we saw the free use of ordinary antiseptic agents, with or without drainage. At the end of that year hypochlorites were introduced, and about a year later the Carrel technique was evolved, soon to be followed by the Bipp method. In 1917 flavine and coloured antiseptic pastes—brilliant green, etc.—appeared, and, generally speaking, their advent marked the end of the pure antiseptic development. Meanwhile, the method of wound excision, ardently supported since early 1916 by many surgeons, steadily gained ground, and a combination of the whole culminated in what may be termed the period of primary wound suture. Realisation had by then become general that antiseptics were inefficient without a careful and extensive cleansing from the wound of all damaged, dead, or non-viable tissue, and thenceforward developed the phase of selection, standardisation, and application of appropriate combined methods to all varieties of wounds encountered.

The many successful results obtained by immediate excision before bacteria had had time to penetrate deeply into the neighbourhood of the wound suggested, as Bowlby points out, that the period elapsed since receipt of the injury must be of the greatest importance. To a large extent delay is dangerous, but this applies more to the actual cleaning up of the wound than the suturing itself. If the cases of lesser injury be taken first, one sees that in 1917 the French, who were probably the pioneers in this field, reported that 80 per cent. of lighter wounds without fracture were successfully closed within eight to twelve hours after being received. Also, in dealing with more severe cases they found that, provided the primary operation was immediately undertaken, that scrupulous asepsis was observed, and the greatest care taken to remove all hopelessly damaged tissue, but at the same time to preserve as much as possible of the normal parts, that then the procedure of "delayed primary suture" might be employed with astonishing success. In fact, it was stated that forty-eight hours or more delay may be actually advantageous in allowing more complete purification. Bowlby, from his own experiences, agrees in saying that "no definite rule can be laid down as to the lapse of time after which suture should not be done; but the sooner the wound can be operated upon the greater the probability of success. It can be sutured later."

Thus in 1918 was the method commended, but much was left to the surgeon's judgment.

### Some General Indications.

It was advised that doubtful wounds, very extensive lacerations, compound fractures, etc., were best left open for a day or two after the first operative measures until time has elapsed to show what sepsis was to be expected, and to show the most advisable course to be taken. Immediate attention, however, at least reduced the probability of extending infection and suppuration, and Tissier, with whom a great deal of the recent advance originated, has demonstrated that, if immediate cultures from a wound do not show either streptococci or the pathogenic anaerobes, then immediate primary suture may be performed. He and Gross advocate, in fact, that, after moderate surgical cleansing, immediate routine primary closure should be adopted, and, unless streptococci be detected in the early discharges, the original stitches should be left *in situ*. If, however, the streptococcus appears, then the wound should be reopened and a removal of further tissue performed to check the septic process. Others have decided the bacteriology before closure, but worked upon the same principle, and their published figures demonstrate well above 80 per cent. of success.

There are, however, certain definite limitations to such methods. They are not applicable in emergencies; they require all the essentials of good aseptic technique and are advisedly to be carried out in a hospital where the man can remain fourteen days at least and be under the observation of the

**War and Surgical Progress—(*continued*).**

same set of medical officers throughout. Given such conditions, even then records indicate that immediate primary suture should not be attempted in very large wounds with deep irregular tracks, in those accompanied by great shattering of bone or unreachable foreign bodies, or in any major lesions older than eighteen hours. The use of radiography, stereoscopy, electric localisation, and a host of accessory agencies has been largely employed by the Americans and ourselves to bring more of such cases within the possibility of immediate closure methods, but there still remain a considerable number upon which the risk is too high for justification. With good fortune, however, it may in these be possible to perform either "delayed primary suture," if sepsis does not intervene, or "secondary suture" after a period of antiseptic treatment with bacteriological control.

### Position of Antiseptic Methods.

The advent of primary wound suture in no sense throws such methods into a class with the obsolete. They have their definite value as indicated, but the fact is now appreciated that in an enormous number of wounds, if seen and operated upon early, such laborious dressings and technique are unnecessary. Essentially they remain treatments for deeply infected wounds, and since 1915, when the early deplorable stage of overwhelming contamination was passed, they have daily relapsed more and more into the position of a reserve power in wound surgery.

Of course, the extent of their decline from general application varies considerably in different hands and in different schools. Barnsby, after experience with several long series of cases, suggests that primary suture should be performed only upon simple articular wounds or lesions of joints with only slight bony injury, upon supra-aponeurotic wounds of soft parts, and if sub-aponeurotic, only those the floor of which can be plainly seen after incising.

Even in these, if asepsis is at all uncertain or the lesion is of more than twenty hours' standing, he emphasises the need of methods such as the Carrel-Dakin before closure is justified.

On the other hand, we have the more enthusiastic views of the French. In their support one may instance the report by Lemaitre in 1918, who recorded primary suture, immediate or delayed, in 2,537 cases, with seventy-nine per cent. of successful results, and this in a list of injuries comprising: 2,030 wounds of soft parts (1,060 very serious and 250 associated with bony, vascular or nervous injury); eighty-seven injuries of large joints; 263 wounds with diaphysial fractures; 110 wounds of hand or foot with major injury; forty injuries of skull and brain; and seven of the thorax.

### In Future.

All who went through in the early days in Flanders, before the application of at least secondary wound suture had become a reality, know how large a number of wounded had to be evacuated before any semblance of true healing had occurred. Applied experience has now brought us to a stage in which the majority of casualties require little more than a passive supervision of the process of repair. It is sometimes unbelievable, after the horrors of 1914, that most of the recently injured men had their stitches removed in 12 to 15 days, and could be evacuated to base hospital or on leave, according to the severity of their condition, within three weeks. Such statements may appear exaggerated, but it must be remembered that one does not refer to the obviously difficult cases, but to the total wounded sent back. Obviously, spreading infection, gas gangrene, bad general condition with shock, shattered limbs needing amputation, severe lesions of blood-vessels, etc., are all absolute contra-indications and require to be viewed from an entirely different standpoint. Discussions of improvement in their treatment we must leave, for we are here concerned with generalisation alone.

Undoubtedly the procedure of primary suture has come to stay. It has been proved applicable in the stress of war; how much more so, among the advantages of the homeland hospital where the three essentials—a skilled surgeon, perfect equipment, and time—are ready to hand. The surgeon has much of his old days treatment of casualties to revise, but the War has given us many who by now have learnt the lesson, and that in the sternest of schools.

